# Association between perceived partner support and lifestyle in mother-father dyads expecting a first child

**DOI:** 10.3389/fpubh.2022.912768

**Published:** 2022-09-06

**Authors:** Vickà Versele, Annick Bogaerts, Roland Devlieger, Christophe Matthys, Leonardo Gucciardo, Tom Deliens, Peter Clarys, Dirk Aerenhouts

**Affiliations:** ^1^Department of Movement and Sport Sciences, Faculty of Physical Education and Physiotherapy, Vrije Universiteit Brussel, Brussels, Belgium; ^2^Department of Development and Regeneration, Faculty of Medicine, KU Leuven, Leuven, Belgium; ^3^Faculty of Medicine and Health Sciences, Centre for Research and Innovation in Care, University of Antwerp, Antwerp, Belgium; ^4^Faculty of Health, University of Plymouth, Plymouth, United Kingdom; ^5^Obstetrics and Gynaecology, University Hospitals Leuven, Leuven, Belgium; ^6^Department of Obstetrics, Gynaecology and Fertility, Leuven, Belgium; ^7^Department of Chronic Diseases and Metabolism, Clinical and Experimental Endocrinology, KU Leuven, Leuven, Belgium; ^8^Department of Endocrinology, University Hospitals Leuven, Leuven, Belgium; ^9^Department of Obstetrics and Prenatal Medicine, Faculty of Medicine, Vrije Universiteit Brussel, Brussel, Belgium; ^10^Obstetrics and Prenatal Medicine, University Hospital Brussel, Brussels, Belgium

**Keywords:** pregnancy, physical activity, dietary intake, parents, couples, social support

## Abstract

**Background:**

A healthy lifestyle during pregnancy is important for the health of mother and child. However, unfavorable physical activity (PA) and dietary changes are observed in pregnant women and their partner. Partner's influence on lifestyle has been reported by expectant women and men. The first aim was to analyze associations between perceived partner support on expectant parents own as well as their partner's moderate-to-vigorous intensity PA (MVPA) levels and dietary intake. Secondly, this study aimed to investigate intra-couple associations of MVPA, dietary intake and perceived support.

**Methods:**

A total of 152 heterosexual couples were recruited between week 8 and 10 of gestation by means of convenience sampling. Objective anthropometric and 7-day MVPA measurements were performed at 12 weeks of gestation. An online questionnaire was used to assess dietary intake, perceived partner support and socio-demographic characteristics. An Actor-Partner Interdependence Model for distinguishable dyads was constructed to examine the relationship between perceived partner support and both own's (i.e., actor-effect) and the partner's (i.e., partner-effect) MVPA levels, intake of fruits and vegetables, and an “avoidance food group.”

**Results:**

For pregnant women, perceived support from their partner was significantly associated with women's own MVPA levels (i.e., actor-effect; estimate = 0.344, SE = 0.168, *p* = 0.040) as well as the MVPA levels of the men (i.e., partner-effect; estimate = 0.717, SE = 0.255, *p* = 0.005). No significant actor- nor partner-effects were found for the expectant fathers. For none of the sexes significant actor-effects were found for fruit/vegetables and “avoidance food group” intake. For pregnant women, there was a positive partner-effect for fruit and vegetable intake (estimate = 7.822, SE = 1.842, *p* < 0.001) and a negative partner-effect for the “avoidance food group” intake (estimate = −16.115, SE = 3.629, *p* < 0.001). Positive correlations were found for perceived MVPA support (*r* = 0.40, *p* < 0.001), MVPA levels (*r* = 0.24, *p* = 0.007) and food intake from the “avoidance food group” (*r* = 0.28, *p* = 0.005) between partners.

**Conclusion:**

This study shows that male partners can act as significant facilitators for women. Partners may be an important target when promoting MVPA during pregnancy. Additionally, supportive couples seem to strengthen each other in keeping a healthy lifestyle in early pregnancy. These results justify couple-based interventions in the promotion of a healthy lifestyle during the transition to parenthood.

**Clinical trial registration:**

Clinicaltrials.gov, identifier: NCT03454958.

## Introduction

Adequate physical activity (PA) and a healthy diet during pregnancy are important for the health and wellbeing of both mother and child (1, 2). The World Health Organization (WHO) advises adults to engage in moderate-to-vigorous intensity PA (MVPA) for at least 150 min per week, with additional health benefits when reaching 300 min per week (3). This advice also applies to complication-free pregnant women. Recommendations about the quality and quantity of the maternal diet during pregnancy mainly comprise adequate macro- (i.e., protein, fat, complex carbohydrates) and micronutrient (e.g., folate, iron, iodine) intakes, with a specific focus on nutrient-dense foods and an overall balanced energy intake (4, 5). On top of generally recognized health benefits, MVPA and a healthy diet during pregnancy are important for the prevention of excessive gestational weight gain, complications during pregnancy (e.g., gestational diabetes), and have been shown to be beneficial for the child (6–8). Moreover, improving PA levels and dietary adequacy have been shown to be useful in the prevention of depression and are related to improvements of pregnant women's overall quality of life (5, 9, 10).

MVPA levels however appear to decrease (too much) throughout the pregnancy period (11, 12). Also dietary intake of women changes during pregnancy (13), and is generally inadequate and not in line with the recommendations (14). It has been shown that the social environment of pregnant women is an important aspect in terms of PA and eating behavior (12, 13, 15, 16). This was recently confirmed by Rockliffe et al., demonstrating that social influences appear to affect women's decision-making about their health behavior (17). Social support can be categorized into four groups of support: (1) emotional support; (2) informational or cognitive support; (3) instrumental support (i.e., assistance received in terms of tangible goods, services or aid) and (4) appraisal support (i.e., information useful for self-evaluation) (18, 19). Professional advice is perceived as important in the promotion of a healthy lifestyle during pregnancy (20) and might be important for informational support. Family and friends on the other hand seem to be a constant source of information and advice throughout pregnancy (21). However, advice from family, friends and partner was often perceived as incorrect and discouraging throughout pregnancy (12, 22–24). Nevertheless, family and friends should be considered as sources of instrumental and emotional support. Partners can for example act as significant facilitators for performing MVPA and obtaining or maintaining healthy eating habits (25–28), and may thus play an important role when promoting a healthy lifestyle during pregnancy. The role of partner support on women's lifestyle during pregnancy has indeed been studied in a qualitative way, but the association between levels of partner support and levels of MVPA and dietary intake has not yet been studied (23).

Not only pregnant women, but also men experience changes in PA and eating behavior when expecting their first child (11–13, 29). The influence of the pregnant partner was described as an important interpersonal determinant explaining (undesirable) changes in expectant fathers' lifestyle during the pregnancy of their partner (12, 13). Even though the importance of social support for fathers and the support fathers need from their partner during interventions to improve their lifestyle has been described (30, 31), studies investigating the influence of partner support on MVPA levels and dietary intake of expectant fathers are still lacking.

The two individuals which constitute a couple can be considered as a simple social system. Instead of studying individuals independently, considering the couple as a pair or dyad takes into account daily interpersonal interactions between both members of the couple (32). Yet, no research has applied a dyadic approach to examine the interdependence of perceived support in terms of MVPA and eating habits within expectant couples and actual MVPA levels and dietary intake. Moreover, pregnancy is considered as a “teachable moment” during which future mothers and fathers are concerned about the health of their baby and consequently are motivated to improve their lifestyle (29, 33–36). Parents (-to-be) also reported a need for couple-based lifestyle interventions to start within pregnancy (37). To this end, the first aim of this study was to analyze if perceived partner support for engaging in MVPA and healthy eating is associated with actual MVPA levels and dietary intake during the pregnancy of a first child, considering a dyadic data structure. It was hypothesized that those parents perceiving more support would engage in more MVPA and show a healthier dietary intake. The second aim was to assess the intra-couple associations of MVPA, dietary intake and perceived support. It was hypothesized that there would be a positive correlation between the health behaviors within the couples.

## Materials and methods

### Design and participants

This study is part of the multi-center longitudinal observational TRANSPARENTS study (Trial registration: Clinicaltrials.gov, NCT03454958) of which the protocol was described in depth elsewhere (38). The overall aim of the TRANSPARENTS study is to gain insight into changes in body weight, body composition and energy balance related behavior (EBRB), i.e., diet, PA and SB, during the transition to parenthood. Baseline data were used for the purpose of the present cross-sectional study. At the obstetrics units of four participating hospitals (University Hospital Leuven, University Hospital Brussel, Jessa Hospital, Hospital Oost-Limburg) in Belgium, a total of 304 expectant parents (i.e., 152 heterosexual couples) were recruited during the first prenatal visit (i.e., between week 8 and 10) of gestation by means of convenience sampling. Around the first routine scan, at gestational week 12 (range ±2 weeks), baseline data were collected at the participating hospitals or the participants' home, depending on their preferences. The Medical Ethics Committee of the University Hospital of Vrije Universiteit Brussel (UZ Brussel, Brussels, Belgium) approved the study protocol and all related documents (B.U.N. 143201835875). The study was conducted in compliance with the principles of the Declaration of Helsinki (current version), the principles of good clinical practice (GCP) and in accordance with all applicable regulatory requirements. All participants signed an informed consent prior to study participation.

### Procedure and measurements

During the study visit at 12 weeks of gestation, anthropometrics were objectively assessed. A calibrated scale (TANITA MC780SMA, Tanita corp., Tokyo, Japan) was used to measure body weight (kg) to the nearest 0.1 kg, and a calibrated SECA wall-mounted stadiometer (1 mm accurate scale) to measure height (m). BMI (kg/m^2^) was calculated by dividing body weight by squared height. The participants moreover received an accelerometer (GT3X+, Actigraph, USA) with instructions concerning the wearing of this accelerometer and on keeping a daily activity log. Participants were asked to wear the accelerometer on the right hip for seven consecutive days, starting the day following the anthropometric measurements, and for at least 12 h/day. Manual data-cleaning of the accelerometer data was done based on the daily logs kept by the participants, which provided information on activities during which the accelerometer was removed (e.g., water activities). Days with accelerometer wear time of <10 h were removed and participants with <5 days of valid accelerometer data were excluded (39). Freedson's cut points were used to calculate time spent in MVPA (intensity ≥3 MET) (min/day) (40).

**Figure 1:**
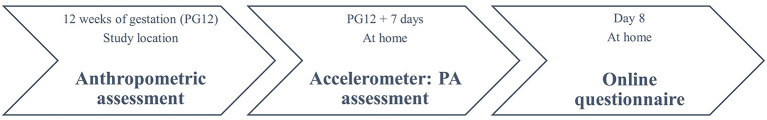
Overview of different steps during data collection. Study location: participating hospitals or participant's home.

An online questionnaire was sent to the participants at the end of the week in which the Actigraph accelerometer was worn. This questionnaire was used to assess dietary intake (i.e., food group intake), perceived partner support in terms of MVPA and eating habits, and socio-demographic characteristics [sex (male/female), age (birthday, from which age at study visit was calculated), net-family income (predefined categories: < € 2,000, € 2,000–€ 3,000, € 3,000–€ 4,000, € 4,000–€ 5,000, >€ 5,000/month)]. A reminder to complete the questionnaire was sent three times with intervals of 1 week. An overview of the timing of the data collection is shown in Figure 1.

A validated 22-items Food Frequency Questionnaire (FFQ) was used to assess average daily intake of different food groups, based on the Belgian Food Based Dietary Guidelines (41, 42). There were two questions for each food group. First, average frequency of intake over the last month was questioned (never, 1–3 days/month, 1 day/week, 2–4 days/week, 5–6 days/week, every day). The answer was recoded to a value that represented a proportion of the average consumptions per day. Hereafter, the average portion per day had to be chosen from a list of predefined portions (in grams accompanied by real life examples) specific for each food group. For each individual food group, the average consumption per day was then multiplied by the average portion per day, which resulted in the daily consumed portion. Based on the different food groups, two new principal food groups were calculated which are indicative for a healthy dietary pattern: (1) daily fruit and vegetable intake (i.e., sum of the “fresh, canned, frozen and dried fruit,” “raw and cooked vegetables” and “soups” food groups); and (2) daily intake of food groups that should be avoided as much as possible (i.e., sum of the “sugary drinks,” “sweet and salty snacks,” “sauces,” “sweet spreads” and “processed meat products” food groups) for which we will refer to as “avoidance food group” intake. Moreover, weighted average energy content of each food group was multiplied with the daily consumed portion, after which total energy intake was calculated by computing the kcal/day intake of all food groups. Finally, as FFQs tend to underestimate dietary intake, daily consumed portions of the two new food groups of interest for this paper (i.e., fruit & vegetables and “avoidance food group”) were divided by the total energy intake and multiplied by 1,000 in order to have the daily intake in g per 1,000 kcal. To assess partner support, the Social Support Surveys for Diet and Exercise Behaviors was used (43). The original and validated social support questionnaire is used to assess support from family (defined as “members of the household”) and friends (43). For the purpose of this study, only support from family was questioned, which was renamed to “support from partner.” A Dutch version of the questionnaire was developed in two steps. In a first step, the questionnaire was independently translated by two researchers (VV and HVE) from English to Dutch. In a second step, the translated questionnaires were compared and differences in translation were discussed. Doubts or disagreements were discussed with another researcher (DA) until consensus was reached (i.e., face-validity). The scoring of the questionnaire was calculated based on the original version's scoring protocol (43). The perceived partner support score in terms of MVPA was calculated by summing all scores on questions about partner participation (i.e., questions 1–6 and 10–13, e.g., “my partner gave me helpful reminders to exercise”) and partner reward (i.e., question 9) and by subtracting scores on questions about partner discouragement (i.e., questions 7 and 8, e.g., “my partner complained about the time I spend exercising”) (43). This resulted in a score ranging from 1 (no experienced support) to 53 (highest possible experienced support). For the perceived social support score for eating habits, scores on questions about encouragement [i.e., questions 1–5, e.g., “my partner encouraged me not to eat “unhealthy foods” (cake, salted chips) when I'm tempted to do so”] were summed, after which scores on questions about discouragement (i.e., questions 6–10, e.g., “my partner brought home foods I'm trying not to eat”) were subtracted (43). This resulted in a total score ranging from −20 (no experienced support) to 20 (highest possible experienced support). The terminology “perceived MVPA support” and “perceived eating support” will further be used to refer to the partner support in terms of MVPA and eating habits, respectively.

### Statistical analysis

**Figure 2:**
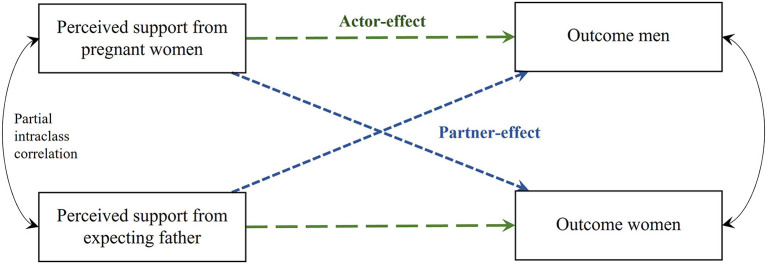
Actor-Partner Interdependence Model of the relation between perceived partner support and outcome. Green dashed line, actor-effect; blue dotted line, partner-effect.

Data were analyzed using R version 4.1.2. Histograms were used to check distribution of MVPA, food groups and sample characteristics, and boxplots were used to check for extreme outliers, which were absent. Mean and standard deviation (SD) were calculated for normally distributed data, and median and interquartile ranges (IQR) for non-normal distributed data. Descriptive statistics and groupwise comparisons (Independent samples *t*-test, Chi^2^-test, Mann-Whitney *U*-test) were done using the package tableone (44). An Actor-Partner Interdependence Model (APIM) for distinguishable dyads (i.e., both members of the dyad can be differentiated from each other, in this case based on their sex) was constructed to examine the relationship between perceived MVPA support and MVPA levels, and between perceived eating support and fruits and vegetables, and “avoidance food group” intake corrected for total energy intake. APIM estimates an actor-effect, this is the extent to which an independent variable of a person (e.g., “perceived partner support in this study”) influences his own outcome of a dependent variable (e.g., “MVPA”). It moreover estimates a partner-effect, this is the extent to which the same independent variable influences the outcome of a dependent variable of his or her partner (i.e., partner-effect) (Figure 2) (45). The Lavaan program and structural equation modeling (SEM) were used for the analysis (46) utilizing the APIM_SEM application (47). Missing data ranged from 0 to 6% across variables, and full information maximum likelihood (FIML) estimations were used to address missingness. FIML is an often used model-based method in SEM analyses and directly estimates parameters at the population level based on all information in the sample data (48). The effect of the perceived partner support on his/her own outcome (actor-effect), and the effect of the perceived partner support on the partner's outcome (partner-effect) were examined. Family income was included in the analyses as a between-dyad covariate, and BMI and age of both parents were included as within-dyad covariates. Partial intraclass correlations were calculated to assess associations between perceived partner support and outcomes within the couple and as effect sizes for actor- and partner-effects. A 5% significance level was used.

## Results

Men were significantly older (men: 31.4 ± 4.1 years, women: 29.5 ± 3.6 years, *p* < 0.001) and had a higher BMI (men: 25.3 ± 3.8 kg/m^2^, women: 24.3 ± 4.8 kg/m^2^, *p* = 0.046) than their female partners. Perceived partner support in terms of MVPA and eating did not significantly differ between women and men (*p* = 0.069 and *p* = 0.863 respectively). More sample characteristics can be found in Table 1.

**Table 1:** Characteristics of the study sample.

	**Men (*n* = 152)**	**Women (*n* = 152)**	***P*-value**
Weeks of gestation [mean (SD)]	12.83 (0.96)		
Household income (%)			
<2,000 e/month	3.4		
2,000–3,000 e/month	6.1		
3,000–4,000 e/month	45.6		
4,000–5,000 e/month	29.9		
More than 5,000 €/month	15.0		
Age in years [mean (SD)]	31.4 (4.1)	29.5 (3.6)	**<0.001** ^a^
BMI in kg/m^2^ [mean (SD)]	25.3 (3.8)	24.3 (4.8)	**0.046** ^a^
Perceived MVPA support [mean (SD)]	20.0 (7.6)	21.7 (8.8)	0.069^a^
Time spent in MVPA in min/day [median (IQR)]	34.6 (22.1, 57.4)	22.9 (13.2, 35.1)	**<0.001** ^b^
Categories of MVPA (%)			**<0.001** ^c^
<150 min/week	22.4	45.1	
150–300 min/week	39.2	39.6	
>300 min/week	38.5	15.3	
Perceived eating support [mean (SD)]	2.0 (4.8)	2.1 (5.4)	0.863^a^
Fruit and vegetable intake in grams/day [mean (SD)]	316 (182)	433 (204)	**<0.001** ^a^
“Avoidance food group” intake in grams/day [median (IQR)]	205 (100, 473)	233 (111, 414)	0.838^b^
Total energy intake in kcal/day [mean (SD)]	1,507 (461)	1,329 (317)	**<0.001** ^a^

BMI, body mass index; PA, physical activity; SD, standard deviation; IQR, interquartile range. ^a^independent samples *t*-test; ^b^Mann-Whitney *U*-test, ^c^Chi^2^-test. Bold values are the significant values.

### Perceived MVPA support and MVPA levels (Model 1)

**Figure 3:**
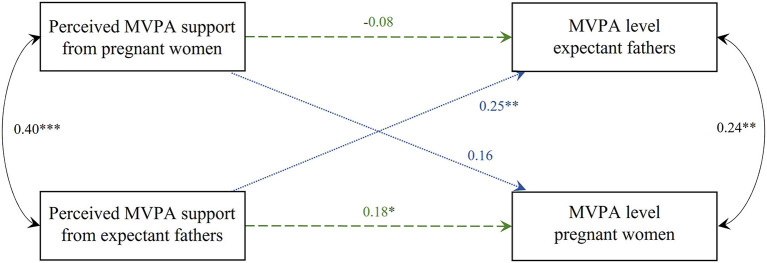
Actor-Partner Interdependence Model of the relation between perceived MVPA support and MVPA levels in women and men. Standardized parameter estimates are shown, *p < 0.05; **p < 0.01; ***p < 0.001. Model corrected for family income (between-dyad covariate), BMI and age (within-dyad covariates). Green dashed line, actor-effect; blue dotted line, partner-effect; MVPA, moderate-to-vigorous physical activity; BMI, body mass index.

The APIM model with the standardized parameter estimates of perceived MVPA support and MVPA levels is shown in Figure 3. For women, perceived MVPA support from their partner was positively associated with their own MVPA levels (i.e., actor-effect; estimate = 0.344, SE = 0.168, *p* = 0.040) as well as the MVPA levels of the men (i.e., partner-effect; estimate = 0.717, SE = 0.255, *p* = 0.005). No significant actor- nor partner-effects were found for men. Family income and age were significant covariates for men's MVPA levels, i.e., the higher the income and the older, the more likely men displayed higher levels of MVPA (estimate = −7.836, SE = 2.291, *p* = 0.001 and estimate = 1.306, SE = 0.519, *p* = 0.012, respectively). BMI was not a statistically significant covariate. The results also showed a significant and positive partial intraclass correlation for MVPA levels between partners (*r* = 0.24, *p* = 0.007); i.e., when one partner scores high (low) on MVPA after controlling for the predictor variables, the other partner also tends to have a high (low) score on MVPA. In addition, perceived MVPA support in women was positively related to men's perceived MVPA support (*r* = 0.40, *p* < 0.001).

### Perceived eating support and dietary intake (Model 2 and 3)

#### Perceived eating support and fruit and vegetable intake corrected for total energy intake (Model 2)

**Figure 4:**
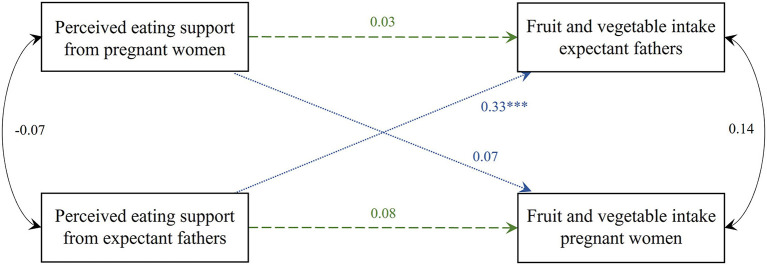
Actor-Partner Interdependence Model of the relation between perceived eating support and fruit and vegetable intake corrected for total energy intake in women and men. Standardized parameter estimates are shown, ***p < 0.001. Model corrected for family income (between-dyad covariate), BMI and age (within-dyad covariates). Green dashed line, actor-effect; blue dotted line, partner-effect; BMI, body mass index.

The APIM model with the standardized parameter estimates of perceived eating support and fruit and vegetable intake is shown in Figure 4. No significant actor-effects were found. There was a positive partner-effect for women's perceived eating support, i.e., a higher score on perceived eating support from their male partner was positively associated with the men's own fruit and vegetable intake (estimate = 7.822, SE = 1.842, *p* < 0.001). This partner-effect was not found in men. None of the covariates included in the model were statistically significant. Partial intraclass correlations for both fruit and vegetable intake and perceived support between partners were not significant (*r* = 0.14, *p* = 0.374 and *r* = −0.07, *p* = 0.107, respectively).

#### Perceived eating support and “avoidance food group” intake corrected for total energy intake (Model 3)

**Figure 5:**
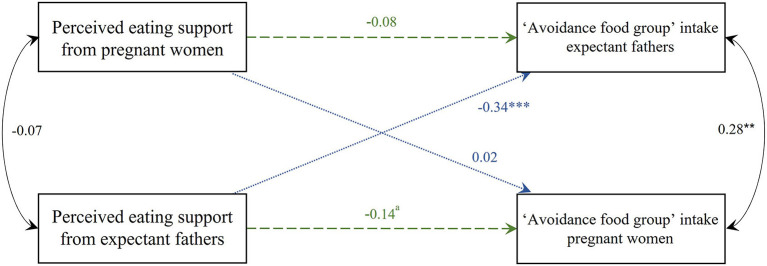
Actor-Partner Interdependence Model of the relation between perceived eating support and “avoidance food group” intake corrected for total energy intake in women and men. Standardized parameter estimates are shown, **p < 0.01; ***p < 0.001, ^a^trend toward significance *p* = 0.077. Model corrected for family income (between-dyad covariate), BMI and age (within-dyad covariates). Green dashed line, actor-effect; blue dotted line, partner-effect; BMI, body mass index.

The APIM model with the standardized parameter estimates of perceived eating support and “avoidance food group” intake is shown in Figure 5. A negative trend toward significance was found for the perceived eating support which pregnant women experienced from their male partner with women's own “avoidance food group” intake (i.e., actor-effect) (estimate = −3.744, SE = 2.116, *p* = 0.077). The perceived eating support from their male partner was negatively associated with the men's own “avoidance food group” intake (i.e., partner-effect) (estimate = −16.115, SE = 3.629, *p* < 0.001). No significant actor- nor partner-effects were found for men. For both women and men, BMI was a significant covariate (estimate = 5.357, SE = 2.611, *p* = 0.040 and estimate = −11.008, SE = 5.083, *p* = 0.030 respectively). Family income and age were no significant covariates. The partial intraclass correlation for “avoidance food group” intake between partners was positively significant (*r* = 0.28, *p* = 0.005); i.e., when one partner scores high (low) on intake from the “avoidance food group,” the other partner also tends to have a high (low) score on intake from the “avoidance food group” after controlling for the predictor variables.

## Discussion

The present cross-sectional study examined the associations between perceived partner support in terms of MVPA and eating habits, and MVPA levels, fruit and vegetable, and “avoidance food group” intake at the end of the first trimester in pregnancy. By conducting Actor-Partner Interdependence Models, it was possible to study the dyadic perspective and support among expectant couples in relation to healthy lifestyle parameters. The novel findings from this study highlight the interdependence of couples when it comes to support and lifestyle.

As hypothesized, the analyses showed that perceived support from the male partners was significantly and positively associated with MVPA levels in pregnant women. In contrast to the hypothesis, perceived support in terms of eating habits was not associated with pregnant women's intake (i.e., no actor-effects) of the food groups studied. This suggests that among pregnant women, higher perceived support is associated with higher MVPA levels and vice versa, but not with higher intakes of fruits and vegetables or lower intakes of “avoidance food group” products. Whether this suggests that PA support is more effective than diet related support, or whether women experienced more PA support compared to diet related support is not clear and cannot be derived from the data.

With median MVPA levels of 35 min/day (i.e., 245 min/week) for men and 23 min/day (i.e., 161 min/week) for women, the recommendations of MVPA per week were met (49). However, 45.1% of participating women and 22.4% of men were below the recommended minimum of 150 min of MVPA per week. As MVPA tends to further decrease toward and similarly after delivery, and because of the clear health benefits of sufficient MVPA during pregnancy, support to obtain or maintain adequate levels of MVPA during pregnancy is of uttermost importance (1, 11, 50). Healthcare providers who are in close contact with parents during the pregnancy period are an important source of support and information (12). Unfortunately, positive enforcement and trusted information is still lacking (22). A study investigating leisure-time PA in women with gestational diabetes showed that more than one fourth of all women reported an absence of encouragement for leisure-time PA from healthcare providers (25). The lack of professional support and regular advice might be attributed to limited time, knowledge and resources available on this topic among healthcare providers (20, 25). Moreover, even though healthcare providers meet with pregnant women on a regular basis, they cannot offer daily or additional support when needed. Male partners could compensate for the limited follow-up offered by healthcare providers. Indeed, as shown by our results, women with more support from their partner engage more in MVPA, and these effects may likely be more pronounced when explicitly targeted in intervention studies. Men can for example be informed about PA during pregnancy which is important to change men's own potential concerns about safety of PA during pregnancy. Consequently, men could also help their pregnant partner to cope with perceptions of risks associated with maternal MVPA and contest incorrect suggestions from the broader social environment (i.e., friends and family) to stop or limit MVPA throughout pregnancy. The partner, with whom one is living, can thus play an important additional supportive role.

Whereas, we did not find an association between partner support and one's own dietary intake, other research showed that women with more partner support in general declared fewer difficulties with healthy eating (51). Nevertheless, comparison with the present study is difficult as in the study of Stampini et al. (51) partner support was not questioned specifically in terms of eating habits and food group intakes but questioned as perceived healthiness of their own eating habits. A plausible reason for not observing significant associations between partner support and dietary intake might be that men go along with the choices of their pregnant partner, even when these choices are unhealthy [e.g., a quote from a recent focus group study (13) “I am not going to say “no” if my wife proposes to order fast food”] (23), or due to their lack of food knowledge (13). Moreover, men sometimes seem to encourage unhealthy eating behaviors (e.g., to eat a double portion for the baby) (13, 23).

Research demonstrated that men want to be involved and are supportive for their partners during pregnancy (23, 52). Men can be encouraging for their pregnant partner to adopt a healthier lifestyle, but this only seems to be the case for health conscious men (23). The important supportive role of partners for engagement in MVPA during pregnancy has been shown by others, especially for leisure-time PA (25, 26). As Actigraphs without additional questions concerning the PA modalities were used in this study, we could not investigate if the perceived support was linked specifically to domain specific MVPA. Confirming our hypothesis, we did find a correlation between MVPA levels within the couple, and research showed that having physically active partners are predictive for engaging in leisure-time PA during pregnancy (25).

The APIM analyses showed that the perceived support women experience from their partner was significantly and positively associated with the men's MVPA level and fruit and vegetable intake, and negatively associated with their partner's “avoidance food group” intake (i.e., partner-effects). This could be explained by the MVPA support score, which partly consists of exercising together (e.g., “*my partner exercised with me,” “my partner changed his schedule so we could exercise together”*). The same is applicable for the eating support score, for which one of the discouragement-related questions was about “*eating fat-, sugar- or salt-rich food products right in front of the partner.”* Thus, pregnant women's perceived support probably relates to their partner's lifestyle.

No associations were found between perceived support from pregnant women and lifestyle behavior of the men, contradicting our formulated hypothesis. Yet, previous qualitative research showed that (at least some) men seem to rely on their partner as main source of information on the topic of a healthy lifestyle during pregnancy (23). Expectant fathers indicated that support from their partner is important to encourage them to adopt a healthy lifestyle (37). But the influence of the pregnant partner was also experienced as a determinant with a rather unfavorable influence on changes in lifestyle of the male partner (12, 13). The current quantitative data could not confirm these qualitative findings. Support for fathers might nonetheless be important, and couples should work together to amplify the effect of supporting each other in adopting a healthy lifestyle. It needs further investigation to explore how and to what extent women might motivate their male partners in terms of healthy lifestyle. Intervention studies are needed to test whether partner support is influential to EBRB of men.

These results are a starting point for the further investigation and for the development of new strategies in lifestyle interventions among expectant couples. Mutual support of both partners might be a way toward behavior change or maintenance of pre-existing healthy behaviors. If healthcare providers are aware of the important supportive role male partners may have concerning the MVPA levels of their pregnant partner, participation in maternity care visits may be encouraged. This would be an opportunity to engage male partners and their support to ensure continuity and follow-up on provided lifestyle advice. It is currently still difficult for men to support their partner in terms of PA, given that they do often not know which exercises are acceptable during pregnancy (23). Men's understanding of the importance of exercise for maternal and fetal health seems to be limited, which often results in beliefs that it is safer to avoid PA (12, 23). The few existing interventional studies (in terms of EBRB) during which involvement of the partner was included indeed showed promising results. Adding aspects of partner support in interventions with the aim to encourage health-related behaviors might be effective in improving partners' awareness, knowledge and self-efficacy (53, 54). A behavior change technique which was described to be useful in a pregnant population is goal setting (55). Goal setting is not an individual and independent activity but should be embedded in social relationships, considering shared goals for both partners of the couple (56, 57). This approach of setting shared goals would be a way to motivate both parents toward success in improving EBRB during pregnancy (23). Collaborative implementation of target goals and planning together are effective in promoting healthy eating and PA (58, 59). The beliefs of a joint effort in achieving goals, and increases of a partner's understanding and support were motivations for women to engage in couple focused interventions (23). Indeed, dyadic lifestyle interventions have a positive effect on PA and SB outcomes in comparison with individual interventions (60). Health education for couples should thus focus on correcting inaccurate perceptions of the risks and health benefits associated with lifestyle during pregnancy and on shared goal setting. The Transactive-goal-dynamics (TGD) theory is a perspective which could be used to conceptualize goal setting within and across relationships (56). Even though expectant fathers can play an important supportive role, they also experience a need for support from their pregnant partner (31).

### Strengths and limitations

A strength of the present study is the use of objectively measured MVPA data of 1 week of both pregnant women and expectant fathers. Most studies use subjective measures of PA, but data from questionnaires in this population is often under- or overreported (61, 62). Objectively measured PA provides more reliable insights into actual levels of PA in this population (63).

The application of dyadic analyses is unique in this population and offers new insights. Nevertheless, the cross-sectional design precludes causal interpretations. Longitudinal analysis investigating changes in social support over the course of pregnancy and in the postpartum period is recommended for future research. Secondly, a validated FFQ was used to assess dietary intake in order to limit the burden of participants compared to other more time-intensive methods. However, the used FFQ was developed based on the national Food Based Dietary Guidelines and validated (41, 42). Distribution of intakes (e.g., total energy intake) was checked revealing no implausible intakes. Moreover, intake of the food groups studied were corrected for total energy intake (expressed as g/1,000 kcal.day) to anticipate on misreporting. Thirdly, participants from this study were volunteers, which resulted in a self-selection bias. Our sample consisted of mainly Caucasian, mostly higher educated healthy participants [e.g., 54.9% of women and 77.7% of men meeting the PA recommendations (49), and fruit and vegetables consumption was higher in comparison with women and men in Belgium in the same age group of 18–39 years old (i.e. 250 and 213 g/day respectively) (64, 65)] and included only heterosexual couples. Our findings may therefore not be generalized to specific (e.g., same-sex couples, parents-to-be with underlying health disorders) or vulnerable populations (e.g., teenage couples, couples with lower socio-economic status) or families with more children.

## Conclusion

Perceived partner support has a positive influence on MVPA levels but not in terms of dietary intake of pregnant women. This perceived support among pregnant women was also positively associated with male partner's MVPA level and fruit and vegetable intake, and negatively associated with male partner's “avoidance food group” intake. No associations were found between perceived support from pregnant women and lifestyle behavior of the expectant fathers. These results indicate that male partners are important sources of daily support and may act as facilitators for improving MVPA in pregnant women. Although future research is warranted to confirm our results and to clarify how expectant fathers can be involved, the use of couple-focused interventions appears to be key for promising outcomes.

## Data availability statement

The dataset used for this study can be retrieved through the corresponding author on reasonable request.

## Ethics statement

The studies involving human participants were reviewed and approved by the Medical Ethics Committee of the University Hospital of Vrije Universiteit Brussel (UZ Brussel, Brussels, Belgium) approved the study protocol and all related documents (B.U.N. 143201835875). The patients/participants provided their written informed consent to participate in this study.

## Author contributions

VV, AB, RD, LG, TD, PC, and DA contributed to the conception and design of the study. VV recruited and measured the participants, was responsible for data extraction, data analyses, and wrote the original manuscript. AB, RD, CM, LG, TD, PC, and DA revised the manuscript critically. All authors were responsible for reflection, interpretation of the data, had full access to all data, have read, and approved the final version of the manuscript.

## Funding

This research was supported by a research grant from the Research Foundation—Flanders [Fonds voor Wetenschappelijk Onderzoek (FWO)] with project number G033418 N. RD is holder of a FWO Fundamental Clinical Investigatorship with number 1803311 N.

## Conflict of interest

The authors declare that the research was conducted in the absence of any commercial or financial relationships that could be construed as a potential conflict of interest.

## Publisher's note

All claims expressed in this article are solely those of the authors and do not necessarily represent those of their affiliated organizations, or those of the publisher, the editors and the reviewers. Any product that may be evaluated in this article, or claim that may be made by its manufacturer, is not guaranteed or endorsed by the publisher.

## Abbreviations

APIM: Actor-Partner Interdependence Model
BMI: body mass index
FFQ: food frequency questionnaire
FIML: full information maximum likelihood
IQR: interquartile range
MET: metabolic equivalents
MVPA: moderate-to-vigorous physical activity
PA: physical activity
SEM: structural equation modeling
SD: standard deviation.
